# Identification of disulfidptosis-related genes in immunity and immunotherapy in diabetic foot ulcer

**DOI:** 10.1097/MS9.0000000000003859

**Published:** 2025-10-08

**Authors:** JIe Li, Hongshuo Shi, Yemin Cao

**Affiliations:** a Diagnosis and Treatment Center of Vascular Disease, Shanghai TCM-Integrated Hospital, Shanghai University of Traditional Chinese Medicine, Shanghai, China; b Department of Peripheral Vascular Surgery, Shuguang Hospital Affiliated to Shanghai University of Traditional Chinese Medicine, Shanghai, China

**Keywords:** diabetic foot ulcer, disulfidptosis, immune infiltration, machine learning, molecular clusters

## Abstract

**Background:**

Diabetic foot ulcer (DFU) is one of the most common and severe complications of diabetes, with vascular changes, neuropathy, and infections being the primary pathological mechanisms. Disulfidptosis, a recently identified form of programmed cell death, might be involved in the development of diabetic complications. This study aims to identify and validate potential disulfidptosis biomarkers associated with DFU through bioinformatics and machine learning analysis.

**Methods:**

We downloaded two microarray datasets related to DFU patients from the Gene Expression Omnibus (GEO) database, namely GSE134431, GSE68183, and GSE80178. From the GSE134431 dataset, we obtained differentially expressed Gln-metabolism-related genes (deDRGs) between DFU and normal controls. We analyzed the correlation between deDRGs and immune cell infiltration status. We also explored the relationship between DRG molecular clusters and immune cell infiltration status. Notably, we used Weighted Gene Co-expression Network Analysis (WGCNA) to identify differentially expressed genes within specific clusters. We used Gene Set Variation Analysis (GSVA) to explore which pathways might be related to the DRGs. Subsequently, we constructed and screened the best machine learning model. Finally, we validated the predictions’ accuracy using a nomogram, calibration curves, decision curve analysis, and the GSE80178 and GSE68183 datasets.

**Results:**

In both the DFU and normal control groups, we confirmed the presence of deDRGs and an activated immune response. From the GSE134431 dataset, we obtained 33 deDRGs, including MYH10, MYL6, UBASH3B, SLC7A11, DSTN, CD2AP, ME1, OXSM, NDUFC1, GYS1, SCO2, NLN, HNRNPH2, MRPS17, SART3, SAFB2, SAFB, HNRNPU, HNRNPM, MYH14, GTF2I, MYH3, CNOT1, PCBP2, GLUD1, MYH11, TLN2, CHD4, SQSTM1, NDUFB11, NDUFS2, SAMM50, and PPIH. Furthermore, two clusters were identified in DFU. Immune infiltration analysis indicated the presence of immune heterogeneity in these two clusters. Additionally, we established a support vector machine model based on five genes (RALY, R3HCC1, CES1, TCEAL3, and F13A1), which exhibited excellent performance on the external validation datasets GSE80178 and GSE68183 (AUC = 1).

**Conclusion:**

This study has identified five disulfidptosis genes associated with DFU, revealing potential novel biomarkers and therapeutic targets for DFU. Additionally, the infiltration of immune-inflammatory cells plays a crucial role in the progression of DFU.

## Introduction

Diabetic foot ulcer (DFU) is a severe complication of diabetes. Non-healing and persistent inflammation are hallmarks of chronic diabetic ulcers and significant factors contributing to the difficulty of DFU healing. Among approximately 150 million diabetes patients worldwide, 15%–20% develop foot ulcers, and 40%–80% of these patients experience ulceration combined with diabetic foot infections^[1]^. Non-healing DFU generates a significant socioeconomic burden, estimated to cost $4 billion annually, with the cost of each amputation surgery potentially exceeding $53 500^[1]^. In the clinical management of DFU, various approaches are used, including intravenous administration of antibiotics and local treatments such as silver ion dressings, insulin topical application, and anti-inflammatory gels. However, long-term use of these treatments can lead to drug resistance and even delay wound healing^[2]^. Therefore, researching the molecular mechanisms of DFU and developing new therapies that can suppress chronic wound inflammation are crucial for improving treatment outcomes and prognosis for patients with DFU.

**HIGHLIGHTS**

- This study is the first to investigate the role of disulfidptosis-related genes (DRGs) in diabetic foot ulcer (DFU).
- We identified 33 differentially expressed DRGs and characterized their immune infiltration profiles in DFU tissues.
- Patients with DFU were classified into two distinct immune-related molecular subtypes based on DRG expression patterns.
- A support vector machine model comprising five key genes (RALY, R3HCC1, CES1, TCEAL3, and F13A1) achieved perfect diagnostic performance (AUC = 1.0).
- Our results suggest that disulfidptosis and its related genes may serve as novel diagnostic biomarkers and potential therapeutic targets for DFU.

The slow onset and delayed resolution of inflammatory response are among the major factors contributing to the difficulties in wound healing^[3]^. Immune cell infiltration is also a pivotal factor in the onset and progression of DFU. When viewed from a molecular standpoint, the process of wound healing takes place following a disruption in the skin’s protective barrier. This process is typically facilitated by growth factors and cytokines released by specialized cells that become activated as part of the immune response.^[4]^. Savaya and colleagues observed that in cases of DFU, the transcription factors FOMI1 and STAT3, which support the survival of immune cells, are suppressed. This ultimately hinders the process of wound healing in diabetic patients^[5]^. Through this study, our objective is to clarify the potential of immune-related biomarkers in enhancing the effectiveness of DFU treatment.

Disulfidptosis, a novel form of programmed cell death^[6]^, is caused by the excessive accumulation of cysteine within cells, leading to disulfide bond formation. This results in the denaturation of actin filaments and subsequent collapse of the cytoskeleton, leading to cell death^[7]^. From a mechanistic perspective, the high expression of SLC7A11 along with increased glucose consumption leads to excessive disulfide bond formation between cytoskeletal proteins. This further results in the collapse of the actin filament network and subsequent cell death^[7]^. SLC7A11 is a protein that helps cells acquire cysteine and produce glutathione^[8]^. Cysteine and glutathione are crucial for maintaining cellular health and balance^[9]^. Low glucose levels cause SLC7A11 to cease its function and disrupt cellular homeostasis, leading to oxidative stress and an increase in disulfide bonds^[10]^. Disulfide bond denaturation primarily affects the actin cytoskeleton, which is a structure composed of protein filaments that give cells shape and strength^[11]^. Disulfide bonds cause actin filaments to disassemble and aggregate together, leading to cell death. Disulfide bond denaturation differs from other types of cell death as it involves the collapse of disulfide bond proteins and actin. However, currently, there is very little research directly linking disulfidptosis to DFU. This suggests that disulfidptosis holds the potential to become a novel therapeutic target^[12]^.

**Figure 1.**
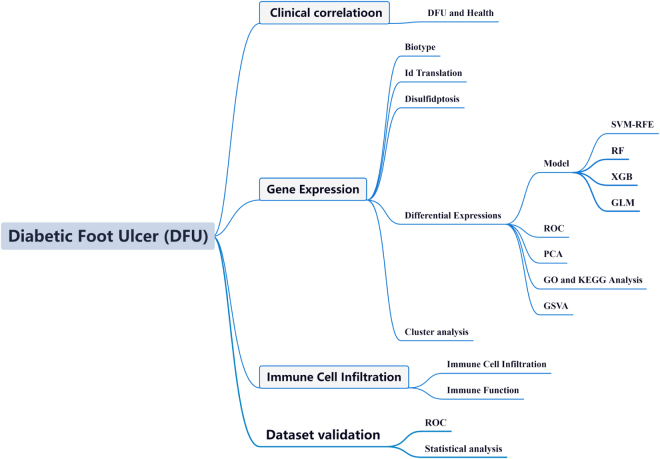
Flowchart of this study.

Nonetheless, the exact mechanisms underlying the pathogenesis of DFU remain elusive. Hence, we propose that disulfidptosis-related genes (DRGs) play a significant role in DFU development^[13]^. In this study, the diagnostic model of DFU-related DRGs can serve as a biomarker for disease diagnosis and treatment monitoring, as well as a reference for early treatment targets of DFU. The flowchart of this study is illustrated in Figure 1. This study adheres to the TITAN Guidelines 2025 for transparent reporting of traditional knowledge and non-AI-assisted research^[14]^.

## Materials and methods

### Raw data processing

The analysis utilized the Gene Expression Omnibus (GEO) datasets GSE134431, GSE80178, and GSE68183. GSE134431 was used as the training set, while GSE80178 and GSE68183 served as the validation set. The related genes of disulfidptosis are derived from relevant literature^[13]^. The information for all datasets is presented in Table 1.

**Table 1:** Dataset information

Dataset	Platform	Count	DFU	Control
GSE68183	GPL16686	6	3	3
GSE80178	GPL16686	12	9	3
GSE134431	GPL18573	21	13	8

### Analysis of differentially expressed genes

Accurate mRNA data were obtained through Perl matching and sorting of transcript data. After data normalization of GSE134431, GSE68183, and GSE80178, differentially expressed genes (DEGs) were identified using the criteria of FDR < 0.05 and |log2FC| ≥ 1 to assess the changes in DRGs.

### Cluster analysis of DFU patients

We determined the ideal cluster count by assessing a combination of criteria, including the cumulative distribution function curve, consensus clustering score, and consistency matrix. For this study, we established a maximum cluster count of *k* = 9.

### Immune cell infiltration

Immune cell composition in DFU tissue was analyzed using CIBERSORT. We used the limma package to construct barplots and corplots to display the results of immune cells.

### Enrichment analysis

We utilized Gene Ontology (GO) and the Kyoto Encyclopedia of Genes and Genomes (KEGG) to investigate biological functions and pathways. We employed R to assess how the differentially expressed DRGs influence biological processes, molecular functions, and cellular components, with this analysis performed using the Gene Set Variation Analysis (GSVA) method.

### Co-expression gene identification

The Weighted Gene Co-expression Network Analysis (WGCNA) algorithm is used to classify genes and identify relationships between modules and features. A co-expression network was constructed using the top 25% variable genes from dataset GSE134431. The dynamic tree-cutting method with a threshold of 0.25 was employed to merge modules. Finally, we mapped the modules with the strongest correlation from the two classification methods.

### Building prediction models based on multiple machine learning methods

The identification of cluster-specific DRGs involves the use of WGCNA and the cross-analysis of DEGs within gene clusters. A Venn diagram is used for visualizing overlapping genes. We used the “caret” R package to build machine learning models based on two different CRG clusters, including support vector machine (SVM), eXtreme Gradient Boosting (XGB), generalized linear model (GLM), and random forest (RF).

### Construction and independent validation analysis of the column line chart model

Using the R package “rms” (version 6.3.0), a column line chart model was constructed, with corresponding scores for each predictive variable. The “Total Score” is the sum of the scores for the predictive variables. Additionally, calibration curve and decision curve analysis (DCA) were used to estimate the predictive ability of the column line chart model. The external datasets GSE80178 and GSE68183 were used to validate the model’s ability to differentiate between DFU and normal controls. Furthermore, the R package “pROC” was used to visualize the receiver operating characteristic (ROC) curve.

### *Drug*–*gene interactions*

In the realm of disease diagnosis, the progress in bioinformatics has underscored the growing significance of creating biological models and pinpointing efficacious biomarkers. Nonetheless, the utilization of these biomarkers in clinical contexts remains of paramount importance. Predicting drug responses using informative markers is imperative for the future prevention and treatment of DFU. The utilization of the DGIdb database helps predict drug–gene interactions for cross-genetic and generated central genes in the RF model, enabling accurate drug prediction and providing references for therapeutic interventions.

## Results

### Expression of DRGs in DFU patients

**Figure 2.**
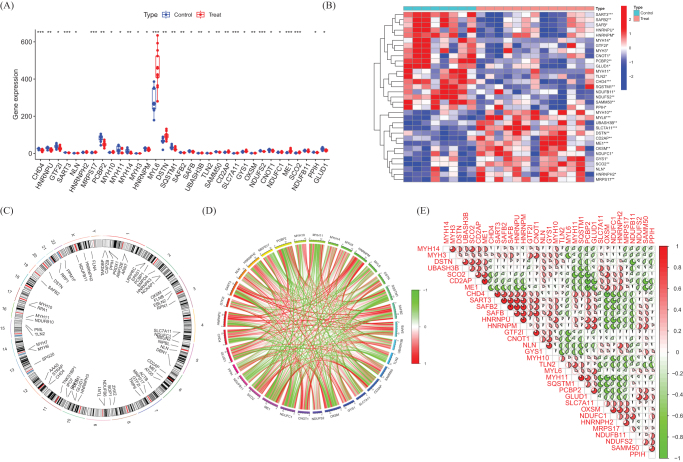
Identification of deDRGs in DFU. (A) The expression levels of DRGs. (B) Heatmap of deDRGs. (C) The location of DRGs on chromosomes. (D) Gene relationship network diagram of deDRGs. (E) Correlation analysis of deDRGs. Red and green colors represent positive and negative correlations, respectively. The correlation coefficient was expressed as the area of the pie chart.

We identified 33 differentially expressed DRGs (deDRGs). Among them, MYH10, MYL6, UBASH3B, SLC7A11, DSTN, CD2AP, ME1, OXSM, NDUFC1, GYS1, SCO2, NLN, HNRNPH2, and MRPS17 showed higher expression levels in DFU testicular tissue. In contrast, the expression level of SART3, SAFB2, SAFB, HNRNPU, HNRNPM, MYH14, GTF2I, MYH3, CNOT1, PCBP2, GLUD1, MYH11, TLN2, CHD4, SQSTM1, NDUFB11, NDUFS2, SAMM50, and PPIH in DFU testicular tissue was significantly lower than those in normal controls (Fig. 2A, B). The chromosomal positions of the DRGs were calculated and visualized in the form of circles (Fig. 2C). Subsequently, we performed correlation analysis on these genes (Fig. 2D, E). Most of these genes showed positive correlations with each other.

### Immune infiltration analysis

**Figure 3.**
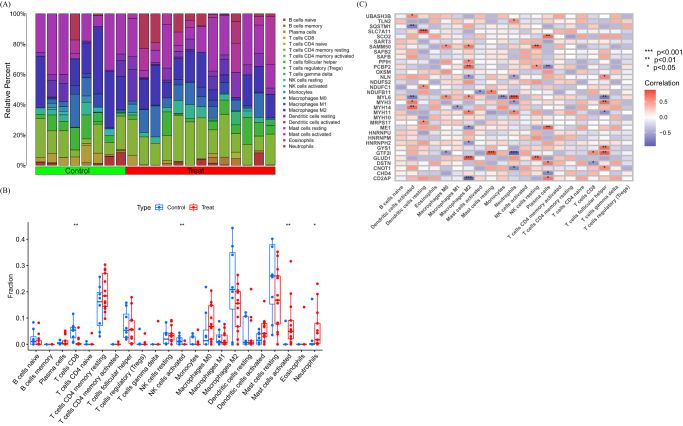
Expression of immune cells. (A, B) Expression of immune cells in different clusters. (C) Correlation between DRGs and immune cells.

The immune environment plays a critical role in the occurrence and progression of DFU. The distribution of immune cells in various samples is shown in Figure 3A. The immune cell differences between the DFU and control groups are depicted in Figure 3B. In DFU patients, we found more activated mast cells and neutrophils, while NK cells and CD8+ T cells were less common. The correlation analysis between immune cells and DRGs is shown in Figure 3C.

### Cluster analysis

**Figure 4.**
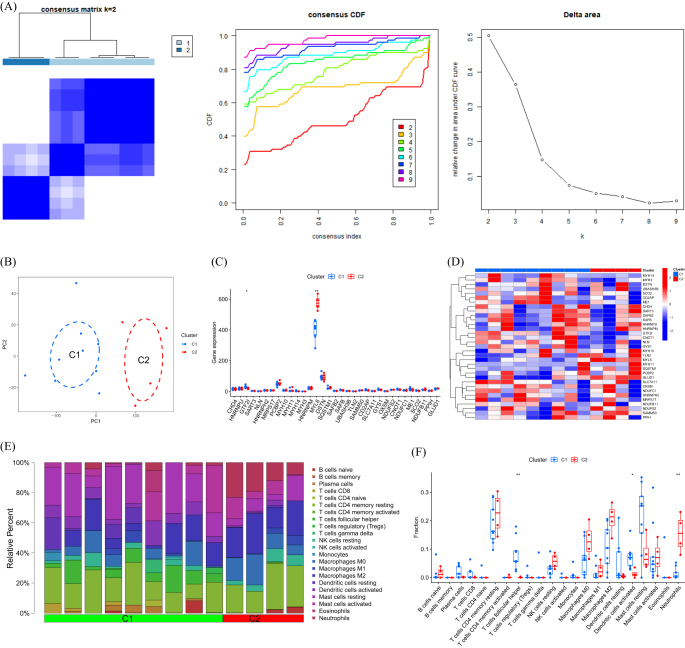
Identification of DRG clusters in DFU. (A) Consensus clustering matrix when *k* = 2. (B) PCA visualized the distribution of the two clusters. (C) Boxplots of DRGs expressed between the two clusters. (D) Heatmap of the expression patterns of the DRGs between the two clusters. (E) Relative abundance maps of 22 infiltrating immune cells between the two clusters. (F) Boxplots of immune infiltration differences between the two clusters.

When *k* is set to 2, the highest within-group correlation is observed, indicating that DRGs can be used to divide DFU patients into two groups (Fig. 4A). Figure 4B demonstrates significant differences in principal component analysis (PCA) between these two clusters. We also examined the expression of DRGs in different clusters based on this cluster analysis. The levels of CHD4 and MYL6 were found to have significant differences between the two groups (Fig. 4C, D). Furthermore, we conducted an analysis of immune cell infiltration by considering the clusters (Fig. 4E, F).

### Functional enrichment analysis

**Figure 5.**
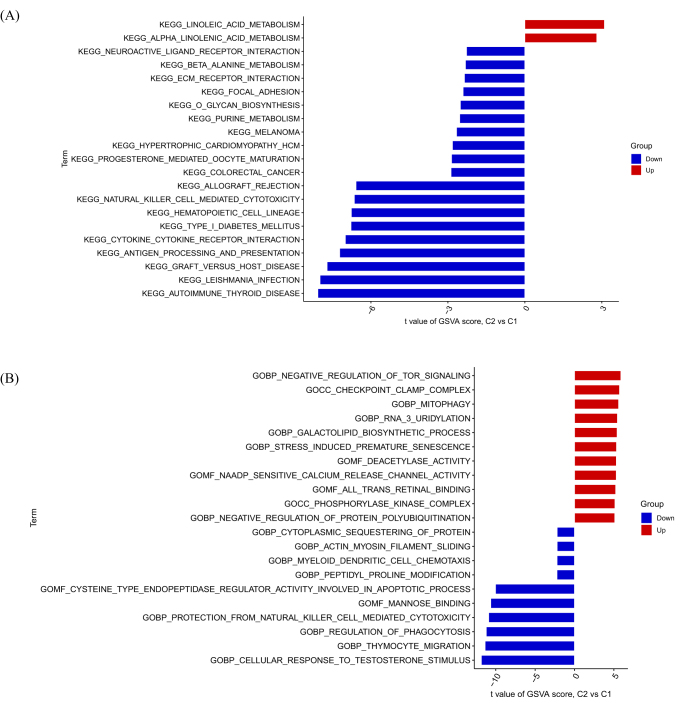
Enrichment analysis for DRGs. (A) KEGG. (B) GO.

Performing GSVA enrichment analysis using DRGs. The pathway is mainly enriched in linoleic acid metabolism, alpha linolenic acid metabolism, autoimmune thyroid disease, and KEGG *Leishmania* infection (Fig. 5A). The GO analysis results include thymocyte migration, cellular response to testosterone stimulus, and negative regulation of TOR signaling (Fig. 5B).

### Gene module selection and construction of co-expression networks

**Figure 6.**
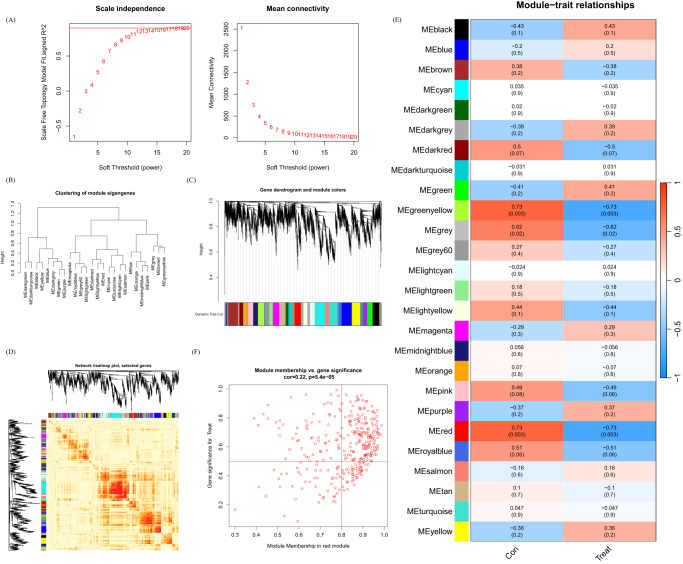
Co-expression network of DEGs in DFU. (A) Set soft threshold power. (B) The cluster tree dendrogram of co-expression modules is shown in different colors. (C) Cluster diagram of module eigengenes. (D) TOM heatmap of 26 modules. (E) Heatmap of correlation analysis of module eigengenes with clinical features. Rows and columns represent modules and clinical features, respectively. (F) Scatter plot of the genetic significance of the blue module members with DFU.

We have built a co-expression network for both normal control and DFU patients using WGCNA and identified key gene modules associated with DFU. We identified the co-expression gene modules under this condition (Fig. 6A). Following that, the dynamic cut algorithm yielded a total of 26 co-expression modules, each represented by distinct colors, and generated a TOM (topological overlap matrix) heat map (Fig. 6B–D). In addition, by applying the genes from these 26 modules, we analyzed the similarity and continuity of co-expression between module clinical features (normal control and DFU). We found that the red module had the strongest association with DFU, which included 222 hub genes (Fig. 6E). Furthermore, there is a positive correlation between the red module and the module-related genes (Fig. 6F).

**Figure 7.**
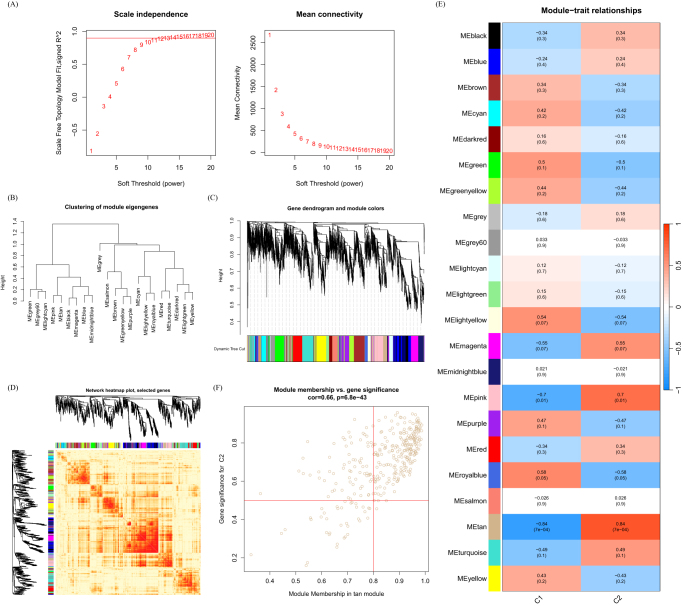
Co-expression network of DEGs between the two cuproptosis clusters. (A) Set soft threshold power. (B) The cluster tree dendrogram of co-expression modules is shown in different colors. (C) Cluster diagram of module eigengenes. (D) TOM heatmap of 22 modules. (E) Heatmap of correlation analysis of module eigengenes with clinical features. Rows and columns represent modules and clinical features, respectively. (F) Scatter plot of the genetic significance of the turquoise module members with Cluster 1.

Furthermore, we analyzed key gene modules closely associated with disulfidptosis clusters using WGCNA. When the soft threshold parameter β is set to 12 and *R*^2^ is 0.9, a scale-free network is constructed under this condition (Fig. 7A). Twenty-two modules were identified as important. The heatmap displays the TOM of all module-related genes (Fig. 7B–D). The examination of the association between modules and clinical characteristics (Cluster 1 and Cluster 2) underscored the importance of the tan module (Fig. 7E). Furthermore, the correlation analysis results indicate a significant positive correlation between the tan module and its corresponding HUB genes (Fig. 7F).

### Construction of the model

**Figure 8.**
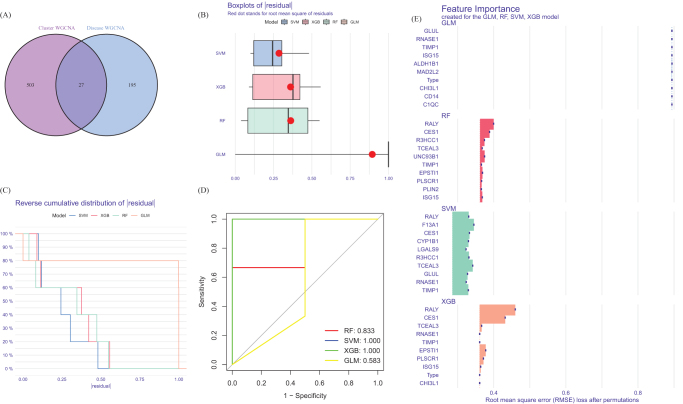
Construction of SVM, RF, XGB, and GLM machine models. (A) Crossover genes of the cuproptosis clusters module and the DFU module. (B) The cumulative residual distribution of the four models. (C) Residual Boxplots of the four machine learning models, where the red dots indicate the root mean square of the residuals. (D) ROC analysis of four machine learning models with 5-fold cross-validation in the test set. (E) The important features in SVM, RF, XGB, and GLM.

We compared genes from the disulfidptosis and DFU modules and found 27 genes that were specific to the disulfidptosis clusters (Fig. 8A, B). The residual distribution results of the four models indicate that RF has the highest residual. Figure 8C ranks the top 10 significant feature variables for each model based on root mean square error. The ROC results of the four machine learning models indicate that the AUC value of SVM is 1.000 (Fig. 8D). Therefore, selecting SVM model (RALY, R3HCC1, CES1, TCEAL3, and F13A1) (Fig. 8E) as the best model is because we believe it can best differentiate between different patient groups.

### Evaluating machine learning models

**Figure 9.**
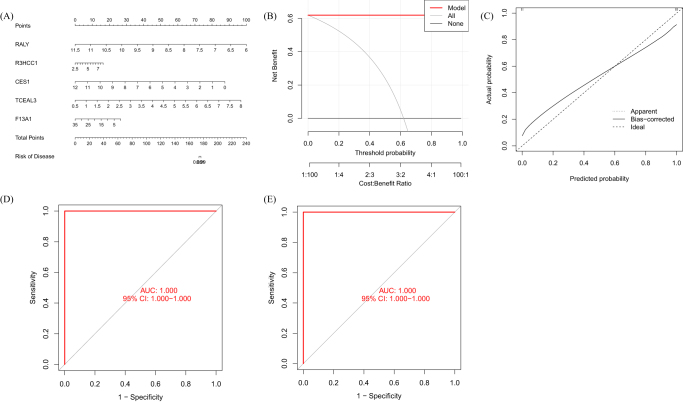
Validation of a five-gene-based SVM model. (A) Construction of a nomogram to predict DFU risk based on a five-gene SVM model. (B, C) Calibration curves. (D) ROC of the five-gene-based SVM model (GSE80178). (E) ROC of the five-gene-based SVM model (GSE68183).

We generated line plots to assess the predictive accuracy of the SVM model (Fig. 9A). The calibration curve and DCA were used to evaluate the predictive accuracy of the line plot model. The calibration curve shows the minimum error between the actual DFU cluster risk and the predicted risk (Fig. 9B). Furthermore, DCA demonstrates that the line plot has high accuracy and can provide reference for clinical decision-making (Fig. 9C). Subsequently, we used the validation datasets GSE80178 (Fig. 9D) and GSE68183 (Fig. 9E) to validate the model, and the ROC result showed an AUC of 1.000, indicating perfect discrimination.

### *Drug*–*gene interactions*

All interacting genes were used for drug prediction (Table 2). F13A1 (coagulation factor XIII) interacted with aspirin, an antiplatelet agent relevant to diabetic vascular complications. CES1 (carboxylesterase 1) showed interactions with 11 drugs, including chemotherapeutics (irinotecan, capecitabine), antihypertensives (enalapril, trandolapril), and anticoagulants (clopidogrel, dabigatran) (Table 2). These predictions highlight repurposing opportunities for DFU adjuvant therapy targeting disulfidptosis-related pathways.

**Table 2:** Drug prediction

Match_term	Match_type	Gene	Drug
CES1	Definite	CES1	CLOPIDOGREL
CES1	Definite	CES1	TRANDOLAPRIL
CES1	Definite	CES1	IRINOTECAN
CES1	Definite	CES1	DABIGATRAN
CES1	Definite	CES1	ENALAPRIL
CES1	Definite	CES1	DIACETYLMORPHINE
CES1	Definite	CES1	CAPECITABINE
CES1	Definite	CES1	ELDACIMIBE
CES1	Definite	CES1	PACTIMIBE
CES1	Definite	CES1	METHYLPHENIDATE
CES1	Definite	CES1	7-ETHYL-10-HYDROXYCAMPTOTHECIN
F13A1	Definite	F13A1	ASPIRIN

## Discussions

DFU represents a complex condition that is unlikely to be effectively addressed using a solitary medication or other intervention measures. The focus of modern drug therapy strategies is to accelerate the healing of chronic wounds and prevent the recurrence of DFU^[14]^. Over the last three decades, our comprehension of the molecular underpinnings of DFU has notably expanded. This has underscored the potential of biomarkers for the development of diagnostic tools, risk assessment, clinical trials, targeted therapies, and the discovery of novel drug targets^[15]^. In this study, we have explored the potential of DRGs as a biomarker in DFU, aiming to contribute to the diagnosis, detection, and treatment of DFU.

Disulfide bonds, arising from oxidative-reduction reactions between two cysteine residues within proteins, are the primary covalent bonds of significance. They are regarded as crucial cellular redox regulators and are intimately linked to disulfide formation^[16]^. In recent times, a novel cell death model named disulfidptosis has emerged. It is triggered by disulfide bond stress and exhibits clear distinctions from the extensively studied types of regulated cell death^[17]^. Within the cell death model, an excess buildup of intracellular disulfide molecules results in disulfide stress. These molecules then bind to actin cytoskeletal proteins, ultimately leading to the disruption of the actin network and subsequent cell demise^[7]^. Studies have shown that elevated SLC7A11 expression limits NADPH production under glucose-deprived conditions. This limitation leads to the excessive accumulation of small-molecule disulfide bonds and a cascade of oxidative-reduction abnormalities, ultimately culminating in cell death. Moreover, NADPH can counter disulfidptosis by disrupting the disulfide bonds within actin cytoskeletal proteins. Prior research has demonstrated that the cortical actin cytoskeleton is not merely a passive observer in single-cell wound repair; instead, it plays an active and dynamic role. This is because nearly all mechanisms involved in plasma membrane repair rely directly or indirectly on the reconfiguration of the cortical actin cytoskeletal framework^[18]^. Therefore, disulfidptosis also has potentially significant implications for DFU, although there is currently a lack of relevant research in this area.

Using the SVM algorithm, we identified five central DRGs (RALY, R3HCC1, CES1, TCEAL3, and F13A1) and validated their diagnostic ability using an external dataset, indicating their potential impact on the pathogenesis of DFU. RALY, a member of the heterogeneous nuclear ribonucleoprotein (hnRNP) family, functions as an RNA-binding protein^[19]^. The primary function of RALY is predominantly associated with its role as an RNA-binding protein. One notable example of this functionality is its involvement in the reprogramming of mitochondrial metabolism through the mediation of miRNA processing^[20]^. Additionally, RALY has the capacity to enhance cell proliferation by overseeing the stability and splicing of specific target mRNAs. Currently, RALY has primarily shown its positive roles in cancer treatment. However, there is currently a lack of related research on its involvement in DFU. The R3HCC1 gene is responsible for encoding a protein comprising the R3H domain and a coiled-coil region referred to as coiled-coil containing 1. This protein is believed to possess nucleic acid-binding properties. In particular, the R3H domain has the capacity to selectively bind to single-stranded DNA and RNA in a sequence-specific manner^[21]^. CES1 is part of the broad serine esterase enzyme family, which is widely distributed among mammals and characterized by its substantial size^[22]^. CES1 is highly abundant in metabolically active tissues such as the liver, white adipose tissue, and brown adipose tissue^[23]^. CES1 plays a crucial role in lipid metabolism and systemic energy homeostasis by catalyzing the hydrolysis of ester bonds and thioester bonds in lipids. This enzymatic activity is observed both *in vitro* and *in vivo*^[22]^. FXIII, a critical player in blood clotting, is a member of the transglutaminase enzyme family^[24]^. The FXIII-A gene (F13A1) is expressed in cells of the bone marrow and mesenchymal lineage^[24]^. Approximately 15%–30% of patients with FXIII deficiency may experience impaired wound healing^[25]^. Studies on mice with FXIII-A deficiency have shown that the healing time of incision wounds is prolonged, and tissue repair is delayed. This can be corrected by administering FXIII concentrate in mice^[25]^. Research has shown that FXIII-A contributes to wound healing and tissue repair through its multifunctional roles^[26]^, starting from its critical function in the hemostatic cascade from platelet adhesion to subendothelium. This process occurs independently of transglutaminase activity and is mediated by integrins αIIbβ3 and αvβ3 located on the surface of platelets^[26]^.

According to research, DFU wounds exhibit characteristics such as high glucose levels, the accumulation of advanced glycation end-products, hypoxia, and ischemia. There is also a significant presence of leukocytes, macrophages, neutrophils, and lymphocytes in the local wound microenvironment. Under the complex interplay of numerous cytokines, this ultimately results in an unfavorable immune microenvironment that hinders the rapid repair of DFU wounds^[27]^. Recently, the immune-inflammatory microenvironment has been identified as one of the reasons for the failure of DFU treatment^[28]^. The pathological features of an abnormal immune microenvironment in DFU primarily involve changes in the types and functions of immune cells, persistent presence of pro-inflammatory factors leading to sustained tissue damage, and local abnormal cell proliferation. This is responsible for the occurrence of chronic inflammation or acute exacerbations of chronic inflammation at DFU sites, which are fundamentally different from pure acute inflammation. Neutrophils are part of the innate immune system and undergo apoptosis after performing their functions at the wound site. Macrophages eventually phagocytose apoptotic neutrophils, providing a strong signal for the resolution of inflammation^[29]^. Neutrophils also play a crucial role in vascular formation and maturation. However, in the disrupted immune microenvironment of DFU, coupled with improper extracellular matrix regulation and the pro-inflammatory environment induced by high glucose and hypoxia at the wound site, neutrophils are continuously recruited. Excessive neutrophils hinder vascular formation and maturation, leading to altered vascular permeability and impaired function. This compromised vascular function fails to provide effective microcirculation, weakening wound contraction^[30]^. The abnormal immune-inflammatory microenvironment leads to impaired NK cell and T cell function, hinders granulation tissue formation, exacerbates inflammation, prevents keratinocytes from migrating and properly epithelializing the wound, delays tissue maturation, and results in the development of chronic non-healing wounds. To successfully treat DFU immune inflammation and restore a normal biological microenvironment at the wound site, both basic and clinical research on DFU must explore unique biomarkers from the perspective of immune-inflammatory interactions.

Our findings on DRGs provide novel mechanistic links to established DFU pathologies. Critically, DFU microenvironments are characterized by hypoxia and glucose fluctuations, conditions known to induce disulfidptosis via SLC7A11 dysfunction and NADPH depletion. The significant upregulation of SLC7A11 in DFU tissues (Fig. 2A, B) suggests impaired cystine metabolism under ischemia, potentially exacerbating disulfide stress and cytoskeletal collapse in resident cells. This aligns with the observed immune dysregulation: sustained neutrophil infiltration and impaired NK/CD8+ T cell function (Fig. 3B) may reflect chronic infection amplified by disulfidptosis-mediated barrier disruption – actin denaturation could compromise phagocytosis and neutrophil extracellular trap formation. Furthermore, chronic hyperglycemia (the hallmark of diabetes) may prime cells for disulfidptosis by altering redox balance through metabolic DRGs like ME1 (Fig. 2B), while neuropathy-driven ischemia creates localized niches for disulfide bond accumulation. Although enrichment of pathways like “autoimmune thyroid disease” (Fig. 5A) lacks direct DFU relevance, these may share upstream immune regulators activated in DFU’s inflammatory milieu. Collectively, DRGs converge on DFU’s triumvirate of hypoxia, infection, and metabolic dysfunction, positioning disulfidptosis as an amplifier of chronicity.

To explore translational potential, we predicted drug–gene interactions for key DRGs using DGIdb. Notably, F13A1 (a coagulation factor) showed interaction with aspirin, a commonly prescribed antiplatelet drug in diabetic patients. CES1 (a metabolic enzyme) exhibited interactions with multiple drugs, including the chemotherapeutic irinotecan and the antihypertensive enalapril. These associations suggest repurposing opportunities for DFU adjuvant therapy.

Research on biomarkers in the context of DFU is still relatively limited. Recently, bioinformatics analysis has become a valuable tool for exploring the intricate and complex connections between cell apoptosis and DFU^[31]^. A comprehensive study has indicated and identified potential biomarkers associated with DFU through transcriptomics and proteomics bioinformatic models, and a study has shown that CXCL11, DDX60, IFI44, and IFI44L are key hub genes with the potential to serve as molecular targets for immunotherapy in DFU^[32]^. However, there are currently very few predictive model studies on cuproptosis and DFU. This study, based on the research of cuproptosis mechanisms, provides a reference for identifying effective immunotherapies in the treatment of DFU. First, we collected comprehensive data on DRGs from continuously updated GEO databases to expand upon early research. Second, GSE134431 was used as the primary dataset for analysis, and it was combined with GSE80178 and GSE68183 to validate the general pattern in the model. GO and KEGG analyses, as well as GSVA analysis, added credibility to this study. Lastly, there are currently almost no predictive models for DRGs that can provide specific recommendations for future immunoinflammatory research or treatments based on disulfidptosis interference in DFU.

The observed immune landscape in DFU, characterized by enriched neutrophils and depleted NK cells/CD8+ T cells, is consistent with a state of chronic inflammation and impaired immune surveillance. Although establishing direct causal links between individual DRGs and specific immune cell alterations necessitates functional studies, we postulate potential connections. Disulfidptosis, by causing cytoskeletal collapse in resident skin cells (keratinocytes, fibroblasts, and endothelial cells), could disrupt tissue integrity and barrier function, releasing damage-associated molecular patterns that perpetuate inflammation and neutrophil recruitment. Furthermore, dysregulation of DRGs involved in redox metabolism (e.g., SLC7A11 and ME1) might alter the oxidative microenvironment, potentially affecting the function and survival of immune cells like lymphocytes. The identified correlations between specific DRGs and immune cell fractions (Fig. 3C) warrant further investigation into their potential regulatory roles.

While this study provides a theoretical foundation and research concept, it still has several limitations. First, the data used in the study are sourced from the GEO database, which presents challenges in establishing the quality and reliability of the statistical data. Therefore, to enhance the quality and reliability of the statistical data, we selected GSE134431 as the main dataset and used GSE80178 and GSE68183 for model validation, which had well-defined groupings.

Second, a key challenge is that this study lacks a fundamental understanding of the mRNA-level systems related to disulfidptosis and DFU. Therefore, there is a lack of insight into the underlying mechanisms at play, and future research will need to design basic experiments for further validation. We admit that although CIBERSORT is a widely used tool, the LM22 matrix is mainly developed based on the characteristics of blood-derived immune cells. Its application in skin tissue and the unique inflammatory environment of DFU may introduce some biases, as the transcriptome profile of immune cells may depend on the context. The research results should take this limitation into account. Future studies using tissue-specific features or single-cell RNA-seq can provide more precise characterization.

Third, while the SVM model demonstrated perfect discrimination (AUC = 1.000) on both the training and external validation sets, this exceptional performance warrants caution. The observed AUC of 1.0 on independent datasets may reflect optimal alignment with these specific cohorts, yet cannot preclude overfitting risks given limited sample sizes. Rigorous validation in larger, multi-center prospective studies is essential to confirm clinical utility.

Fourth, the primary limitation of this study is its reliance on bioinformatic analysis of publicly available datasets. We lack experimental validation of the differentially expressed DRGs and the diagnostic signature in independent clinical samples or relevant cellular/animal models of DFU. Future work will focus on (1) validating the expression levels of the key DRGs, especially the five-gene signature (RALY, R3HCC1, CES1, TCEAL3, and F13A1), in prospectively collected DFU and control tissues using molecular techniques; (2) exploring the functional roles of these genes in regulating disulfidptosis, immune responses, and wound healing processes using *in vitro* (e.g., keratinocyte, fibroblast, and immune cell cultures under high glucose/stress) and *in vivo* (e.g., diabetic mouse wound models) approaches; and (3) investigating the potential of targeting these genes or the disulfidptosis pathway for DFU therapy.

Finally, the publicly available GEO datasets used in this study provided limited or no access to detailed patient clinical metadata. Consequently, we were unable to account for these potential confounding factors or correlate the identified molecular subtypes (clusters) and gene signatures with specific clinical outcomes or phenotypes.

Furthermore, while we identified two distinct DRG-based molecular clusters with differing immune microenvironments, the absence of granular clinical outcome data (e.g., healing time, infection status, amputation risk, and treatment response) in the utilized GEO datasets prevented us from assessing the clinical relevance or prognostic value of these subtypes. Future prospective cohort studies with well-annotated clinical endpoints are crucial to determine whether these immune-heterogeneous clusters correspond to different DFU patient subgroups with distinct clinical behaviors or therapeutic needs.

## Conclusions

The development and progression of DFU result from intricate interactions among diverse targets, signaling pathways, and mechanisms, with regulatory processes that are synergistic and bidirectional. Disulfidptosis might influence how certain genes like RALY, R3HCC1, CES1, TCEAL3, and F13A1, a diagnostic model was established using the correlation between disulfidptosis and these genes. Future improvements involve increasing the quantity of data sources and conducting more scientific and clinical research to determine whether effective treatments can reduce the immunoinflammatory manifestations in DFU patients by modulating the targets and pathways of disulfidptosis. In conclusion, our research results provide broad potential biomarkers for DFU treatment strategies.

## Data Availability

All datasets analyzed in this study (GSE134431, GSE80178, and GSE68183) are publicly available in the GEO database (https://www.ncbi.nlm.nih.gov/geo/).

## Research registration unique identifying number (UIN)

Not applicable (bioinformatic analysis of public datasets).

## Peer and provenance statement

Not commissioned; externally peer-reviewed.
